# Automated external cardioversion defibrillation monitoring in cardiac arrest: a randomized trial

**DOI:** 10.1186/1745-6215-9-36

**Published:** 2008-06-11

**Authors:** Bakhtiar Ali, Heather Bloom, Emir Veledar, Dorothy House, Robert Norvel, Samuel C Dudley, A Maziar Zafari

**Affiliations:** 1 Atlanta Veterans Affairs Medical Center, Decatur, Georgia, USA; 2 Emory University School of Medicine, Division of Cardiology, Atlanta, Georgia, USA; 3 University of Illinois at Chicago, Section of Cardiology and the Jesse Brown VA Medical Center, Chicago, Illinois, USA

## Abstract

**Background:**

In-hospital cardiac arrest has a poor prognosis despite active electrocardiography monitoring. The initial rhythm of approximately 25% of in-hospital cardiopulmonary resuscitation (CPR) events is pulseless ventricular tachycardia/ventricular fibrillation (VT/VF). Early defibrillation is an independent predictor of survival in CPR events caused by VT/VF. The automated external cardioverter defibrillator (AECD) is a device attached by pads to the chest wall that monitors, detects, and within seconds, automatically delivers electric countershock to an appropriate tachyarrhythmia.

**Study Objectives:**

• To evaluate safety of AECD monitoring in hospitalized patients.

• To evaluate whether AECDs provide earlier defibrillation than hospital code teams.

**Methods:**

The study is a prospective trial randomizing patients admitted to the telemetry ward to standard CPR (code team) or standard CPR plus AECD monitoring (PowerHeart CRM). The AECD is programmed to deliver one 150 J biphasic shock to patients in sustained VT/VF. Data is collected using the Utstein criteria for cardiac arrest. The primary endpoint is time-to-defibrillation; secondary outcomes include neurological status and survival to discharge, with 3-year follow-up.

**Results:**

To date, 192 patients have been recruited in the time period between 10/10/2006 to 7/20/2007. A total of 3,655 hours of telemetry data have been analyzed in the AECD arm. The AECD has monitored ambulatory telemetry patients in sinus rhythm, sinus tachycardia, supraventricular tachycardia, atrial flutter or fibrillation, with premature ventricular complexes and non-sustained VT without delivery of inappropriate shocks. One patient experienced sustained VT during AECD monitoring, who was successfully defibrillated (17 seconds after meeting programmed criteria). There are no events to report in the control arm. The patient survived the event without neurological complications. During the same time period, mean time to shock for VT/VF cardiac arrest occurring outside the telemetry ward was 230 ± 50 seconds.

**Conclusion:**

AECD monitoring is safe and likely results in earlier defibrillation than standard telemetry monitoring.

**Trial Registration:**

National Institutes of Health registration ID: NCT00382928

## Background

In-hospital cardiac arrest has a poor prognosis despite active electrocardiography monitoring. Part of the poor prognosis may be explained by slow response to a lethal arrhythmia.[1] The National Registry of Cardiopulmonary Resuscitation (NRCPR) has reported delayed defibrillation, which was defined as greater than 2 minutes, in more than 30% of cases of in-hospital cardiac arrest. This delay in defibrillation resulted in significantly lower probability of surviving to hospital discharge.[1]

The initial rhythm in about 25% of in-hospital cardiac arrest is ventricular tachycardia or fibrillation (VT/VF).[2] Early provision of good quality CPR and rapid defibrillation have the highest impact on survival for the victims of VT/VF cardiac arrest.[3] Early defibrillation is an independent predictor of survival in CPR events caused by VT/VF.[4,5] Delay in provision of defibrillation for 10 minutes renders CPR ineffective.[4] Each minute of delay in defibrillation increases the likelihood of death by 7% to 10% in cardiac arrest.[5] If defibrillation is provided within 3 minutes in in-hospital cardiac arrest, 38% survival to discharge is reported versus 21%, if defibrillation is provided after 3 minutes.[2] Addressing this delay, a program encouraging early defibrillation using automated external defibrillators (AED) in the hospital resulted in a 14-fold increase in survival for VT/VF cardiac arrest.[6]

Automated External Cardioverter Defibrillator (AECD; The PowerHeart CRM, Cardiac Science Inc., Seattle, WA) is a device attached to the chest wall by pads, monitors the electrocardiogram, and is capable of automatically delivering electric countershock to appropriate rhythms without operator intervention. Automated external cardioverter defibrillators have been studied in a few nonrandomized clinical trials. [7-9] They performed with a sensitivity of 100% and a specificity of 98.8% in a study conducted in the United States (n = 79) and a sensitivity of 100% and a specificity of 97.6% in an European study (n = 117).[7,8] We designed the current randomized trial to test the safety of AECDs in hospitalized patients and the performance compared to standard telemetry response.

## Methods

**Figure 1:**
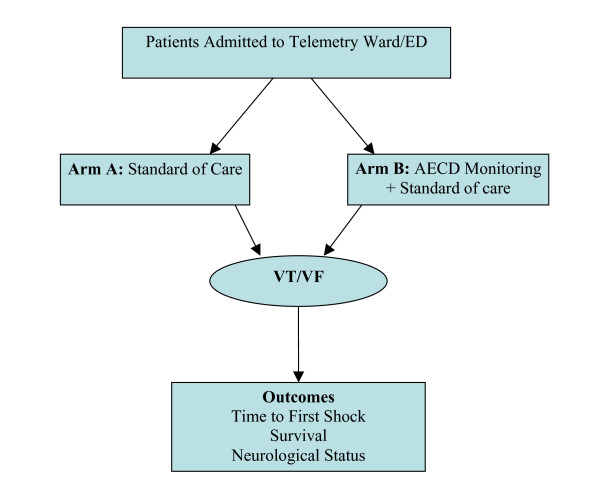
Design of the AECD Trial. ED = emergency department; AECD = automated external cardioverter defibrillator; VT/VF = ventricular tachycardia/ventricular fibrillation.

This study is a single center, randomized, prospective, trial in which, all patients admitted to the emergency department and telemetry unit of the Atlanta Veterans Affairs Medical Center (AVAMC), Decatur, Georgia are screened. Patient recruitment started in October 2006. Inclusion and exclusion criteria are presented in Table 1. Patients consenting to participate in the study are randomized to either standard electrocardiographic telemetry or standard telemetry augmented by AECD monitoring (Figure 1). Patients randomized to AECD monitoring have the AECD attached for the duration of their hospitalization while undergoing simultaneous telemetry monitoring. A log book is used to record the time of AECD attachment and to record whenever the AECD is detached from the patient. The AECD is programmed to deliver a single 150 Joule shock to VT/VF rhythms presenting above the rate of 170 beats per minute, after a 30 second delay. Study enrollment terminates when telemetry is discontinued. The primary endpoint is time-to-defibrillation in VT/VF cardiac arrest. The AECDs have the ability to measure the precise time of initiation of cardiac arrhythmias and of delivery of defibrillation. The secondary outcomes are survival to discharge and cerebral outcomes as measured by cerebral performance category scale (Table 2). Patients who survive cardiac arrest while participating in the trial will be followed for a period of three years for survival, internal cardioverter defibrillator (ICD) placement and cerebral performance. The Emory University Institutional Review Board (IRB) and the AVAMC Research and Development Committee approved the study.

**Table 1:** Inclusion and Exclusion Criteria

**Inclusion Criteria**:
1. All patients admitted to telemetry ward and ER.
2. Age > 18 years.

**Exclusion Criteria**:

1. Pregnant women.
2. Patients with R wave less than 0.5 mV in lead II.
3. Patients with functioning ICDs.
4. Patients with cardiac pacemakers if oversensing by AECD is demonstrated (double counting of pacer spikes).
5. Patients with visible chest lesions that would prevent AECD pad placement.
6. Patients who are designated DNR.
7. Right bundle branch block.
8. Patients with Parkinson's disease.
9. Patients with seizure disorders.

**Additional Exclusion Criteria for Emergency Room**:

10. Patients with dementia and/or delirium.
11. Patients presenting with psychiatric complaints.
12. Patients who are agitated.
13. Patients presenting with trauma.
14. Patients unable to participate in the informed consent process.
15. Patients with respiratory rate greater than twenty.
16. Patients who report pain greater than four out of ten in the visual analog scale.

ER = emergency room; ICD = internal cardioverter defibrillator; AECD = automated external cardioverter defibrillator; DNR = do not resuscitate

**Table 2:** Cerebral Performance Categories/CPC scale

**CPC 1**: Good cerebral performance – conscious, alert, able to work, might have mild neurologic or psychological deficit.

**CPC 2**: Moderate cerebral disability – conscious, sufficient cerebral function for independent activities of daily life. Able to work in sheltered environment.

**CPC 3**: Severe cerebral disability – conscious, dependent on others for daily support because of impaired brain function. Ranges from ambulatory state to severe dementia or paralysis.

**CPC 4**: Coma or vegetative state – any degree of coma without the presence of all brain death criteria. Unawareness, even if appears awake (vegetative state) without interaction with environment; may have spontaneous eye opening and sleep/awake cycles. Cerebral unresponsiveness.

**CPC 5**: Brain death – apnea, areflexia, EEG silence, etc.

EEG = electroencephalogram

### Data collection

Demographic and arrest data is collected according to Utstein guidelines and stored in a database designed with Microsoft access software (Microsoft Inc., Redmond, WA).

### Sample size

A total sample size of 40 patients per group will be recruited. There are approximately 40 true cardiac arrests per year on the telemetry ward. The expected time to defibrillation in the AECD group is to be 30 ± 30 seconds, whereas time to defibrillation in the standard of care group is 180 ± 180 seconds. The expected time to defibrillation in the AECD group will be programmed into the device. Time to defibrillation will be coded continuously in both groups. Using a two-tailed t-test to compare time to defibrillation between the groups, a sample size of 12 patients per group affords 81% power at alpha = 0.05. A total sample size of 40 patients per group will yield over 99% power for a two-tailed test at alpha = 0.05.

### Statistical analysis

The primary outcome will be time-to-defibrillation. Demographic and clinical categorical and continuous variables are compared for patients in the standard of care versus the AECD group at cardiac arrest. Baseline data will be expressed as mean ± SD for continuous variables, and frequencies for categorical variables. Differences in baseline characteristics between the groups will be examined by the use of χ^2 ^tests and two-sample t-tests, or, if assumptions are not met, by Fisher's Exact and Mann-Whitney tests for categorical and continuous variables, respectively. Time to defibrillation will be measured continuously in both the standard of care and the AECD group, then compared using a two-tailed t-test. Assumptions will be checked and analyses will be adjusted accordingly.

In addition a multiple logistic model will be used to analyze the secondary endpoints (survival of cardiac arrest, survival to discharge), considering all the available variables. The total sample size will limit this analysis; however, an exploratory forward stepwise selection procedure will be used with a p-value of at least 0.10 for entry and 0.05 for removal. All pairwise interactions between AECD use and the other variables in the final stepwise model will be tested. A Hosmer-Lemeshow test will be used to test goodness of fit. Odds ratios will be presented with 95% confidence intervals. Pearson residuals, deviance residuals, and influence statistics will be examined to assess model fit. Statistical analyses will be performed using SAS version 9.1.3.

Cerebral performance state (Table 2) will be measured using the cerebral performance categorization (CPC) scale and analyzed initially using χ^2 ^tests. If cell counts warrant, the 2 × 3 contingency table will be analyzed using exact methods and the mean score statistics (Q) will be used to compare cerebral performance status between the groups.

### Preliminary results

One hundred and ninety two patients were recruited in the time period between 10/10/2006 to 7/20/2007. The demographic and clinical characteristics of the patients are presented in Table 3. Patients in the control and treatment arms had similar characteristics. The majority of patients (> 90%) in both arms were men, reflecting the demographics of the AVAMC. A total of 3,655 hours of telemetry data has been analyzed by the AECDs. The AECDs monitored ambulatory patients with normal sinus rhythm, sinus tachycardia, atrial fibrillation or flutter, supraventricular tachycardia, premature ventricular complexes and non-sustained ventricular tachycardia without inappropriately delivering a shock (Table 4). Of the 95 patients randomized to the AECD arm, total of ten patients had to be taken off the AECD, two due to adverse events, two due to anxiety, two due to skin irritation by the pads, and four due to alarming of the AECD, as the pads detached from the chest wall during their sleep.

**Table 3:** Demographic and Clinical Characteristics of Study Subjects

	**AECD **n = 95	**Control **n = 97	**P Value**
**Age ± SD**	61.7 ± 3.4	62 ± 3.4	0.7
**Gender**			0.98
**Male**	92 (96.8%)	94 (96.9%)	
**Female**	3 (3.2%)	3 (3.1%)	
**Race**			0.15
**Hispanic**	2 (2.1%)	0	
**White**	42 (44.2%)	53 (54.6%)	
**Black**	51 (53.7%)	44 (45.4%)	
**History of HF**	25 (26.3%)	20 (20.6%)	0.39
**New Diagnosis of HF**	6 (6.3%)	4 (4.1%)	0.53
**Diabetes mellitus**	46 (48.4%)	35 (36.1%)	0.11
**History of CAD**	35 (36.8%)	42 (43.3%)	0.38
**Hypertension**	79 (84%)	74 (76.3%)	0.28
**Hyperlipidemia**	55 (57.9%)	53 (54.6%)	0.66
**EKG on admission**			0.62
**NSR**	84 (88.4%)	81 (83.5%)	
**Atrial fibrillation/flutter**	9 (9.5%)	11 (11.3%)	
**SVT**	0	2 (2.1%)	
**Other**	2 (2.1%)	3 (3.1%)	

SD = standard deviation; HF = heart failure; CAD = coronary artery disease; NSR = normal sinus rhythm; SVT = supraventricular tachycardia

**Table 4:** Frequency of Abnormal Rhythms Monitored by the AECD

Atrial Fibrillation/Flutter	9 (9.5%)
Supraventricular Tachycardia	1 (1.1%)
Premature Ventricular Complexes	10 (10.5%)
Non-Sustained Ventricular Tachycardia	5 (5.3%)

AECD = automated external cardioverter defibrillator

**Figure 2:**
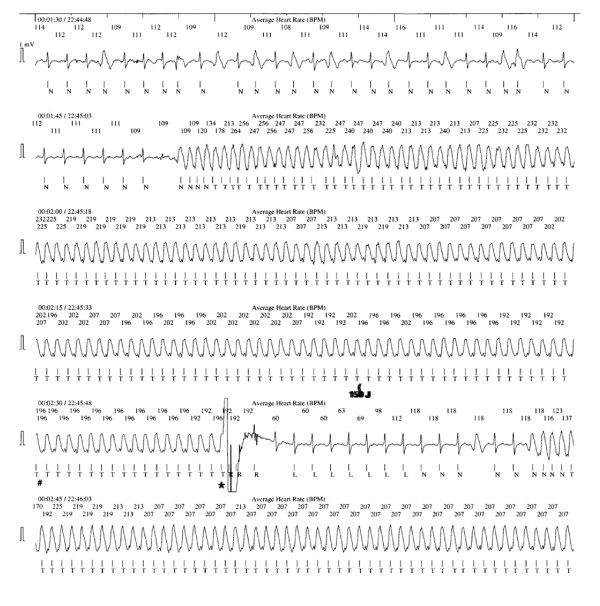
The AECD correctly recognized sustained monomorphic ventricular tachycardia and delivered a 150 Joule shock after 47 seconds. The rhythm is converted to sinus rhythm but reverts to ventricular tachycardia after 10 seconds. (= capacitor charging; # = capacitor charged; * = therapy delivered to patient.

Only one event of sustained VT occurred in the experimental arm, which was defibrillated by the AECD 17 seconds after programmed criteria were met (Figure 2). There are no events to report in the control arm. During the same time period mean time to shock for VT/VF cardiac arrest occurring outside the telemetry ward was 230 ± 50 seconds.

There were two false positive events in which, the shocks were delivered inappropriately. In the first case, the AECD delivered the shock when a patient was eating an apple. The AECD showed ventricular fibrillation at the same time the telemetry monitor showed sinus rhythm. This occurred in the setting of a reduced R wave amplitude on his 12-lead EKG, which was less than 0.5 mV. In the second case, discharge was the result of deliberate action of the patient. Neither patient had any other complications related to the delivery of the shocks.

## Discussion

In-hospital cardiac arrest remains a major health problem with high mortality rates.[2] Most important determinants of survival in both out-of-hospital and in-hospital cardiac arrest are early CPR and defibrillation.[1-3,10-12] Previously three clinical studies evaluated the application of AECD technology in hospitalized patients. [7-9] Mattioni et al. and Martinez-Rubio et al. both demonstrated high sensitivity and specificity for the AECD in the treatment of tachyarrhythmias.[7,8] The two studies demonstrated time-to-shock in the range of 14 to 22 seconds in patients admitted to the intensive care units and the cardiac electrophysiology laboratory.[7,8] In another study in patients with cardiovascular diagnoses admitted to the emergency department, a 94.4% success rate, with a mean time-to-shock of 33.4 seconds for VT/VF arrhythmias was found.[9] These studies, while demonstrating effectiveness of AECDs in highly specialized settings, did not establish efficacy in an ambulatory group, where the possibility of inappropriate discharges are increased. In addition, none of the studies were randomized; there were no control groups with standard of care; and finally, none of the previous studies evaluated the impact of AECDs on survival and cerebral outcomes.

In the one arrest monitored by AECD, we showed a defibrillation time of 17 seconds. This is consistent with previous AECD trials. On the other hand, Chan and his coauthors reported that in 30% of in-hospital cardiac arrest, defibrillation is delayed using standard therapy, resulting in lower survival to discharge (22.2% vs 39.3%, P =< 0.001).[1] After hours (5 p.m. to 8 a.m.), weekends and unmonitored beds were predictors of delayed defibrillation, suggesting that manpower considerations were a critical determinant of response.[1,13] It would seem likely that routine use of automated strategies would help addressing these delays.[14] This idea is supported by findings that AEDs can improve outcomes in in-hospital cardiac arrest.[6]

Our trial thus far, has established the safety and practicality of continuous monitoring by AECDs in a high risk, ambulatory, inpatient population. Having established efficacy, continuation of the trial will allow a comparison of outcomes between the two strategies.

## Abbreviations

AECD: automatic external cardioverter defibrillator; AED: automated external defibrillator; AVAMC: Atlanta Veterans Affairs Medical Center; CPC: cerebral performance categories; CPR: cardiopulmonary resuscitation; IRB: Institutional Review Board; NRCPR: National Registry of Cardiopulmonary Resuscitation; SD: standard deviation; VT/VF: pulseless ventricular tachycardia/ventricular fibrillation

## Competing interests

Dr. Zafari reports receiving an investigator-initiated grant from Cardiac Science, Inc.
